# *RET* fusion–positive non-small cell lung cancer in Sweden: a nationwide registry study

**DOI:** 10.2340/1651-226X.2026.46817

**Published:** 2026-10-29

**Authors:** Ella Äng Eklund, Hans Brunnström, Erik Lampa, Kerstin Gunnarsson, Anders Vikström, Johan Isaksson, Kristina Lamberg, Johan Botling, Henrik Fagman, Sama I. Sayin, Gunnar Wagenius, Levent M. Akyurek, Anders Berglund, Volkan I. Sayin, Andreas Hallqvist

**Affiliations:** a Department of Oncology, Sahlgrenska University Hospital, Gothenburg, Sweden; b Department of Laboratory Medicine, Institute of Biomedicine, University of Gothenburg, Gothenburg, Sweden; c Department of Genetics, Pathology, and Molecular Diagnostics, Skåne University Hospital, Lund, Sweden; d Epistat AB, Uppsala, Sweden; e Department of Oncology, Institute of Clinical Sciences, University of Gothenburg, Gothenburg, Sweden; f Department of Respiratory Medicine, Linköping University Hospital, Linköping, Sweden; g Centre of Research and Development Region Gävleborg, Uppsala University, Sweden; h Department of Medical Sciences, Section of Pulmonology, Uppsala University Hospital, Uppsala, Sweden; i Department of Clinical Pathology, Sahlgrenska University Hospital, Gothenburg, Sweden; j Department of Surgery, Institute of Clinical Sciences, Sahlgrenska Center for Cancer Research, University of Gothenburg, Gothenburg, Sweden; k Wallenberg Centre for Molecular and Translational Medicine, University of Gothenburg, Gothenburg, Sweden; l Department of Oncology-Pathology, Karolinska Institutet, Stockholm, Sweden

**Keywords:** Carcinoma, non-small-cell lung, RET, gene fusion, registries, survival analysis, Sweden

## Abstract

**Background:**

Rearranged during transfection (*RET*) fusion–positive non-small cell lung cancer (NSCLC) is a rare but clinically meaningful molecular subgroup. RET inhibitors have demonstrated marked clinical activity; however, real-world data on disease characteristics and outcomes before their widespread introduction remain limited. This study characterizes clinical features, treatment patterns, and survival outcomes of *RET* fusion–positive NSCLC in a nationwide Swedish cohort, with other driver subgroups as descriptive comparisons.

**Methods:**

Patients ≥18 years diagnosed with histologically confirmed NSCLC in 2018–2022, registered in the Swedish National Lung Cancer Registry and with next-generation sequencing results available, were included. Individuals were categorized by oncogenic driver status, including *RET, ALK, BRAF, EGFR, KRAS, ROS1*, or wild type. Progression-free survival (PFS) and overall survival (OS) from diagnosis were estimated using Kaplan–Meier methods and standardized Cox proportional hazards models adjusted for age, sex, performance status, smoking, stage, and histology.

**Results:**

A total of 68 patients had *RET* fusion–positive NSCLC. The *RET* cohort was characterized by younger age, a high proportion of never-smokers (59%), and predominantly adenocarcinoma histology (96%). Most had stage IV disease at diagnosis and the highest rate of liver metastases (56%). *KIF5B–RET* (71%) and *CCDC6–RET* (19%) were the most frequent fusion partners. Adjusted median OS was 28 months (95% confidence interval [CI]: 13–46) for *RET*, 39 months (95% CI: 29–52) for *ALK*, and 17–26 months for the other groups, with overlapping CIs.

**Conclusion:**

*RET* fusion–positive patients were typically younger never-smokers with adenocarcinoma and had encouraging OS, despite limited access to selective RET inhibitors during the study period.

## Introduction

Non-small cell lung cancer (NSCLC) accounts for approximately 85% of all lung cancer diagnoses and remains the leading cause of cancer-related mortality worldwide [1].

Developments in the diagnosis and treatment of lung cancer during the last decade have led to subgroup categorization based on histopathologic status, transitioning from two main subgroups – NSCLC and small-cell lung cancer (SCLC) – to several molecular subgroups within NSCLC. Currently, these defined subgroups for which there are approved targeted treatments consist of tumors with alterations involving the epidermal growth factor receptor (*EGFR*), anaplastic lymphoma kinase (*ALK*), ROS proto-oncogene 1 (*ROS1*), B-Raf proto-oncogene (*BRAF*), neurotrophic receptor tyrosine kinase 1-3 (*NTRK1-3*), and rearranged during transfection (*RET*), mesenchymal-epithelial transition (MET) exon 14 skipping, Kirsten rat sarcoma viral oncogene (*KRAS*) G12C [2, 3]. In addition to these targetable alterations, programmed death-ligand 1 (PD-L1) expression remains clinically relevant as it is associated with an increased likelihood of response to immunotherapy. Patients who harbor these specific types of fusions/mutations in their tumors benefit to a higher degree from their respective targeted therapies compared to chemotherapy (CT). The patients without targetable mutations usually receive CT and/or immunotherapy [4].

Among the different oncogenic drivers mentioned above, alterations in the *RET* proto-oncogene are one of the more recent fusions that have emerged as an actionable oncogenic driver in a small but clinically distinct subset of patients. RET is normally activated through binding of glial cell line-derived neurotrophic factor (GDNF) ligands and the co-receptor GFRα, leading to dimerization and phosphorylation of downstream signaling proteins, including RAS–MAPK, PI3K–AKT, and JAK–STAT pathways [5]. Oncogenic activation of *RET* in NSCLC occurs predominantly via gene rearrangements (fusions), rather than point mutations or amplifications.

*RET* fusions were first described in lung adenocarcinoma in 2012, initially in the context of tumors lacking other canonical drivers such as *EGFR*, *ALK*, or *KRAS* [6]. These fusions result from chromosomal rearrangements that place the RET kinase domain downstream of constitutively active gene promoters and dimerization domains, most commonly involving *KIF5B–RET* and *CCDC6–RET* [7, 8].

Clinically, *RET*-rearranged NSCLC represents approximately 1–2% of all lung adenocarcinomas [9] and is in general mutually exclusive with other actionable alterations such as *ALK*, *ROS1*, *EGFR*, *BRAF*, or *MET* exon 14 skipping mutations [10].

Until recently, patients with *RET*-altered NSCLC had limited targeted therapy options. Multikinase inhibitors (MKIs) such as cabozantinib, vandetanib, and lenvatinib exhibited modest antitumor activity but were associated with significant off-target toxicities due to non-selective inhibition of VEGFR2, MET, and other kinases [11] and have never been formally approved for this indication. Most *RET* fusion–positive stage IV patients have been treated with standard CT/chemoimmunotherapy in the first-line setting, but the clinical development of selective RET inhibitors, namely selpercatinib and pralsetinib, has changed this management. In the European Union, selpercatinib received conditional marketing authorization in February 2021 and pralsetinib in November 2021. In Sweden, pralsetinib was introduced and reimbursed for *RET* fusion–positive advanced NSCLC in 2022, followed by selpercatinib in 2023; both were reimbursed for patients not previously treated with a RET inhibitor and could therefore be used in the first-line and later-line setting. These agents have demonstrated high response rates, prolonged progression-free survival (PFS), and robust central nervous system (CNS) activity in both treatment-naïve and previously treated patients with *RET* fusion–positive NSCLC [11–13].

Despite the increased interest in *RET* fusion–positive NSCLC patients due to the new targeted therapies, there is still scarce data on this relatively small NSCLC subset with regard to clinical characteristics and prognosis from a population-based perspective. This study aimed to further elucidate clinical characteristics and clinical outcomes among patients in Sweden with NSCLC by *RET* fusion status in relation to other oncogenic driver subgroups and wild-type NSCLC, in patients treated in routine clinical practice prior to the general implementation of selective RET inhibitors.

## Patients and Methods

### Study design

Patients with a diagnosis of NSCLC during 2018–2022 were identified in the Swedish National Lung Cancer Registry (NLCR), a nationwide clinical database that holds information on all lung cancer patients and is linked to the Swedish National Molecular Pathology Registry Data (MolPAT). The patients were divided into separate groups depending on oncogenic driver (RET, ALK, BRAF, EGFR, KRAS, ROS1, or wild type); patients with more than one driver alteration were excluded. In case of missing data for patients with RET fusions in the NLCR, clinical records in the individual patient overview were consulted for registry completion. In the NLCR, data on demographics, diagnostics, treatment, and follow-up (including disease recurrence, new malignant disease, and death) are registered using remote data entry from all Swedish hospital units. The main objectives were to characterize the patient population, analyze the treatment patterns, and assess outcomes regarding PFS and overall survival (OS).

### Patient population

The study population consisted of all patients in Sweden aged ≥ 18 years with histologically confirmed NSCLC registered in the NLCR during 2018–2022 for whom next-generation sequencing (NGS) analyses had been performed.

### Molecular analyses

Molecular analyses of RET, ALK, BRAF, EGFR, KRAS, and ROS1 were performed as part of routine clinical practice at regional molecular pathology laboratories and reported to MolPAT. Depending on the molecular alteration and diagnostic procedures, testing was performed using immunohistochemistry (IHC), in situ hybridization (ISH), and/or NGS. NGS-based testing was introduced in the 2018 national guidelines for non-squamous NSCLC and for squamous cell carcinoma in never- or light smokers and was expanded to include all NSCLC regardless of histology in 2020. RNA-based fusion analysis, including RET, was gradually implemented during the study period. Fusion partners for RET-positive cases were retrieved from the molecular pathology reports.

### Data collection

The following data were collected: clinical characteristics (sex, age, Eastern Cooperative Oncology Group [ECOG] performance status [PS]; smoking status); disease characteristics (date and stage of primary tumor diagnosis and time to relapse/advanced disease, histological subtype, location of metastases); mutational status; treatment strategy (number of treatment lines, therapeutic agents), response and survival status. A treatment line was defined as a new treatment, including possible maintenance delivered for metastatic NSCLC, until progression. The start date of the first-line therapy was defined as the date of the first systemic treatment initiated after diagnosis. Disease relapse/progression was defined as the occurrence of a new metastatic site or progression of existing metastases, regional tumoral or lymph node progression, or discontinuation of systemic treatment due to progression or death from any cause. Relapse and progression were determined by the treating clinicians as part of routine care and recorded in the registry. The NLCR does not capture formal response assessment according to the Response Evaluation Criteria in Solid Tumors (RECIST) version 1.1 [14], but the clinical assessments were based on routine radiological evaluations, which in Swedish lung cancer care are generally reported with reference to RECIST principles. Treatment-related toxicity is not systematically recorded in the NLCR and could therefore not be analyzed. Smoking status was categorized as current, former (> 12 months since smoking cessation), or never smokers, according to the NLCR definitions.

### Statistical analysis

All descriptive statistics were stratified by the presence or absence of the genetic subgroups mentioned previously. OS and PFS were analyzed using the Kaplan–Meier (KM) estimator. For both endpoints, the time origin (index date) was the date of diagnosis for all patients. OS was defined as the time from diagnosis to death from any cause, and PFS as the time from diagnosis to the first recorded relapse/progression or death, whichever came first. Follow-up was truncated at 5 years after diagnosis: patients who were alive (OS) or alive and progression-free (PFS) at 5 years, or at the end of follow-up, were censored. Because the time origin was the date of diagnosis and no grouping in the main analyses was based on events occurring after diagnosis, these analyses are not subject to immortal time bias. OS and PFS were estimated for the cohort as a whole and separately by stage at diagnosis.

Adjusted survival curves were estimated from a multivariable Cox proportional hazards model, adjusting for age, smoking status, stage at diagnosis, sex, ECOG PS, and histology. Predicted survival for each patient was obtained under a fixed exposure level, holding all other covariates constant. These individual predicted survival curves were averaged over the empirical covariate distribution to produce standardized survival curves [15].

Missing data were limited (Table 1). The adjusted analyses were performed as complete-case analyses; that is, patients with missing information on any covariate in the model (ECOG PS, smoking status, or stage) were excluded from the Cox models. For *RET*-positive patients, missing registry data were complemented by review of the medical records where possible.

**Table 1:** Patient characteristics of the study population.

Characteristic *n* (%)	Overall 8,879	RET 68	ALK 315	BRAF 219	EGFR 1,416	KRAS 2,311	ROS1 57	No mutation 4,493
**Sex**								
Male	3,871 (44)	30 (44)	151 (48)	103 (47)	465 (33)	898 (39)	22 (39)	2,202 (49)
Female	5,008 (56)	38 (56)	164 (52)	116 (53)	951 (67)	1,413 (61)	35 (61)	2,291 (51)
**Age at diagnosis [IQR]**	73 [67, 78]	66 [51, 75]	68 [55, 75]	75 [68, 78]	72 [65, 78]	72 [67, 77]	67 [53, 72]	74 [68, 78]
**Age at diagnosis categorical**								
18–60	1,097 (12)	29 (43)	109 (35)	15 (6.8)	242 (17)	241 (10)	23 (40)	438 (9.7)
61–70	2,680 (30)	14 (21)	91 (29)	65 (30)	415 (29)	795 (34)	19 (33)	1,281 (29)
71–80	4,100 (46)	19 (28)	87 (28)	109 (50)	572 (40)	1,090 (47)	8 (14)	2,215 (49)
80+	1,002 (11)	6 (8.8)	28 (8.9)	30 (14)	187 (13)	185 (8.0)	7 (12)	559 (12)
**Smoking status**								
Current	2,764 (31.1)	4 (5.9)	45 (14)	82 (37.4)	137 (9.7)	856 (37)	9 (16)	1,631 (36.3)
Former	4,645 (52.3)	24 (35)	113 (36)	107 (48.9)	621 (43.8)	1,341 (58)	26 (46)	2,413 (53.7)
Never	1,457 (16.4)	40 (59)	157 (50)	29 (13.2)	656 (46.3)	111 (4.8)	22 (39)	442 (9.8)
Missing	13 (0.2)	0	0	1(0.5)	2(0.1)	3 (0.2)	0	7 (0.2)
**Stage**								
I-II	2,508 (28.2)	21 (31)	49 (16)	53 (24.2)	438 (30.9)	678 (29.3)	13 (23)	1,256 (28)
III	1,593 (18)	9 (13)	39 (12)	21 (9.6)	156 (11)	371 (16.1)	4 (7.0)	993 (22.1)
IV	4,724 (53.2)	38 (56)	227 (72)	143 (65.3)	816 (57.6)	1,248 (54)	40 (70)	2,212 (49.2)
Missing	54 (0.6)	0	0	2 (0.9)	6 (0.5)	14 (0.6)	0	32 (0.7)
**Histological subtype**								
Squamous cell carcinoma	1,365 (15)	2 (2.9)	21 (6.7)	18 (8.2)	41 (2.9)	87 (3.8)	5 (8.8)	1,191 (27)
Adenocarcinoma	6,832 (77)	65 (96)	280 (89)	193 (88)	1,337 (94)	2,045 (88)	49 (86)	2,863 (64)
Large cell carcinoma/non-small cell carcinoma unspecified	459 (5.2)	1 (1.5)	8 (2.5)	6 (2.7)	28 (2.0)	139 (6.0)	2 (3.5)	275 (6.1)
Adenosquamous	69 (0.8)	0 (0)	1 (0.3)	0 (0)	5 (0.4)	9 (0.4)	1 (1.8)	53 (1.2)
Sarcomatoid/pleomorphic carcinoma	41 (0.5)	0 (0)	3 (1.0)	1 (0.5)	2 (0.1)	8 (0.3)	0 (0)	27 (0.6)
Unclassified carcinoma	113 (1.3)	0 (0)	2 (0.6)	1 (0.5)	3 (0.2)	23 (1.0)	0 (0)	84 (1.9)
**ECOG performance status**								
0	2,247 (25.3)	28 (41.2)	98 (31.1)	35 (16)	492 (34.7)	593 (25.7)	18 (31.6)	983 (21.9)
1	3,556 (40)	22 (32.3)	126 (40)	89 (40.6)	587 (41.5)	914 (39.5)	22 (38.6)	1,796 (40)
2	1,777 (20)	11 (16.2)	54 (17.1)	52 (23.7)	211 (14.9)	440 (19)	10 (17.5)	999 (22.2)
3	827 (9.3)	2 (2.9)	26 (8.3)	25 (11.4)	89 (6.3)	217 (9.4)	5 (8.7)	463 (10.3)
4	198 (2.2)	0 (0)	6 (1.9)	6 (2.7)	19 (1.3)	59 (2.6)	1 (1.8)	107 (2.4)
Missing	274(3.2)	5 (7.4)	5 (1.6)	12 (5.5)	18 (1.3)	88 (3.8)	1 (1.8)	145 (3.2)
**PD-L1 status (%)**								
Negative < 1	3,173 (35.7)	27 (39.7)	84 (26.7)	55 (25.1)	603 (42.6)	635 (27.5)	13 (22.8)	1,756 (39.1)
Low 1–49	2,527 (28.5)	17 (25)	108 (34.3)	59 (26.9)	391 (27.6)	657 (28.4)	23 (40.4)	1,272 (28.3)
High ≥ 50	2,117 (23.8)	16 (23.5)	76 (24.1)	86 (39.3)	175 (12.4)	708 (30.6)	15 (26.3)	1,041 (23.2)
Missing	1,062 (12)	8(11.8)	47(14.9)	19 (8.7)	247 (17.4)	311 (13.5)	6 (10.5)	424 (9.4)
**Presence of metastasis**	2,161 (24)	16 (24)	102 (32)	60 (27)	376 (27)	550 (24)	21 (37)	1,036 (23)
Liver	804 (37)	9 (56)	51 (50)	27 (45)	144 (38)	181 (33)	11 (52)	381 (37)
Lung	541 (25)	2 (13)	14 (14)	16 (27)	82 (22)	136 (25)	5 (24)	286 (28)
Brain	973 (45)	7 (44)	48 (47)	19 (32)	189 (50)	263 (48)	7 (33)	440 (42)

*n*, number; IQR, interquartile range; ECOG, Eastern Cooperative Oncology Group; PD-L1, programmed death-ligand.

Treatment duration was analyzed using the KM estimator and was assessed in the subset of patients diagnosed with stage IV disease at treatment start. For this analysis, the time origin was the treatment start date, and the time to the event was defined as the number of months from treatment start to treatment stop. Length of available follow-up was estimated using the reverse KM estimator, considering only the time between index and end of the study period.

The survival of *RET*-positive patients with stage IV disease, treated with selpercatinib or pralsetinib, was compared with patients not treated with any of these agents in an exploratory analysis. Exposure to selpercatinib or pralsetinib was treated as a time-varying variable in a multivariable Cox proportional hazards model, where patients could contribute person-time both as unexposed or exposed depending on their current treatment. Person-time before the start of selpercatinib or pralsetinib was thus classified as unexposed, which avoids immortal time bias. Owing to the small number of exposed patients, this analysis was considered exploratory and hypothesis-generating. The model was adjusted for line of therapy, age at diagnosis, sex, ECOG PS, and stage.

All analyses were performed using R, version 4.4.2, using the stdReg add-on package*.*

### Participant consent to release information

This is an observational study that uses patient-approved registry data previously collected and does not impose any form of intervention, and the data have been de-identified to protect participant privacy. Therefore, a formal consent to release information was not required. This study was conducted in accordance with the ethical principles within the Declaration of Helsinki and is consistent with applicable ICH Good Clinical Practice (GCP) Guidelines and applicable laws and regulations in Sweden (The Swedish ethical review authority, registration number DNR2022-06988- 01/DNR2023-05297-02).

## Results

### Study population

A total of 17,812 patients were identified in the NLCR during the selected time period. After exclusion of patients without molecular information or with more than one driver alteration (*n* = 8,473), and of patients with inconsistent dates or incomplete *ROS1* information (*n* = 460) (Figure 1), 8,879 patients were included in the analyses. *RET* fusions were identified in 68 patients, corresponding to 0.8% of all included patients and 1.0% of those with adenocarcinoma (65/6,832).

**Figure 1:**
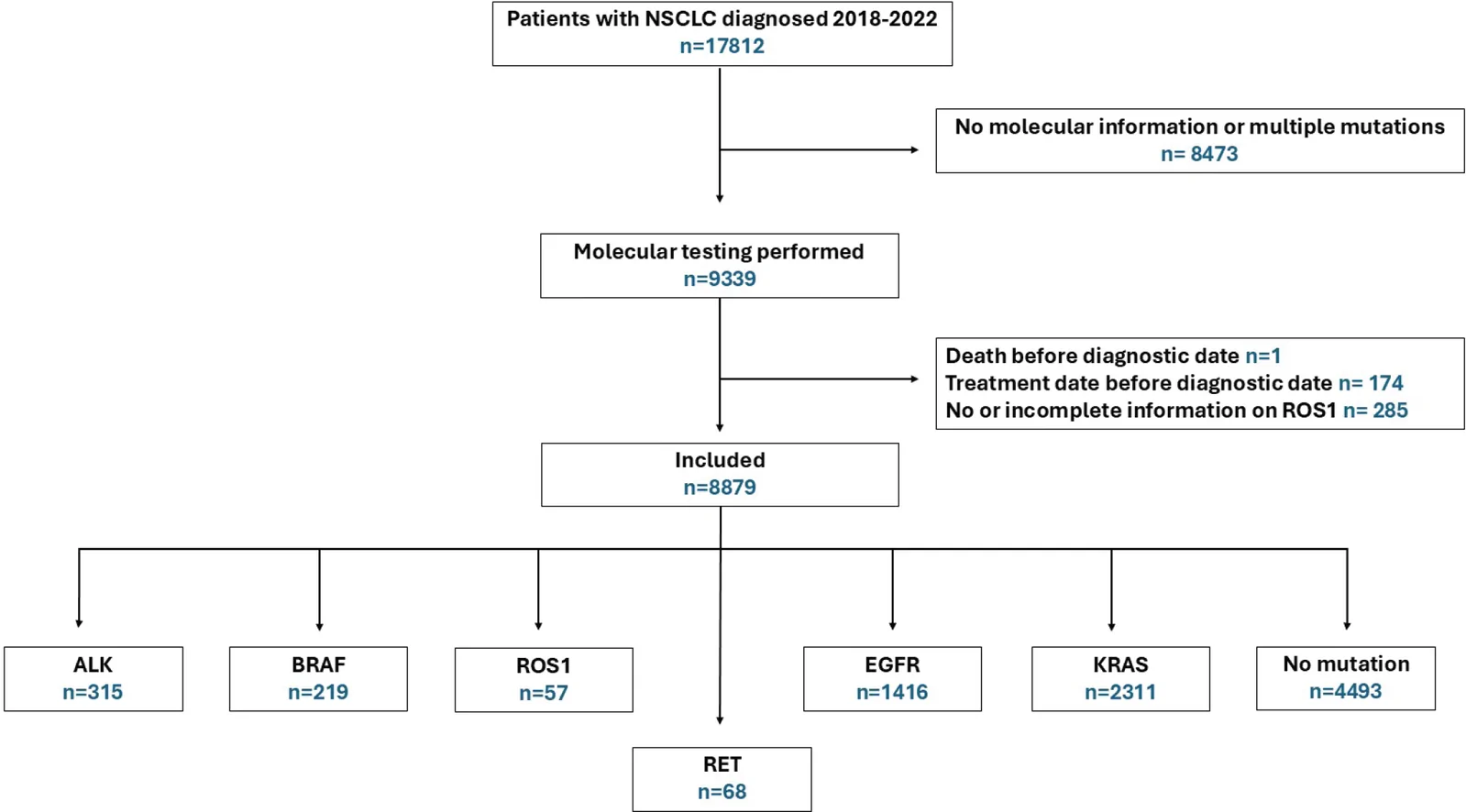
Flowchart illustrating patient selection for the study. NSCLC: non-small cell lung cancer; ALK: anaplastic lymphoma kinase; BRAF: B-rapidly accelerated fibrosarcoma protein; ROS1: ROS proto-oncogene 1; RET: rearranged during transfection; EGFR: epidermal growth factor receptor; KRAS: Kirsten rat sarcoma; No mutation, wild-type *ALK*, *BRAF*, *ROS1*, *RET*, *EGFR*, or *KRAS* genes.

### Patients and disease characteristics

Baseline characteristics for all included patients stratified by molecular driver alteration are summarized in Table 1. The *RET* cohort shows a high proportion of patients diagnosed in the age span 18–60 (43%), a high degree of never smokers (59%), and almost exclusively adenocarcinomas (96%). Three *RET* fusion–positive tumors had non-adenocarcinoma histology: two were classified as squamous cell carcinomas and one as large cell carcinoma/NSCLC not otherwise specified (NOS). The vast majority had an ECOG PS 0-1. PD-L1 status was similar to the overall population. The *RET* cohort had the highest proportion of liver metastases (56%), and the most common fusion partners were *KIF5B* (71%) and *CCDC6* (19%) (Table 2).

**Table 2:** RET fusion partners (n = 68).

Characteristic*n* (%)	68
Fusion partner	
CCDC6	9 (19)
ERC1	2 (4.2)
KIF5B	34 (71)
NCOA4	2 (4.2)
ZNF438	1 (2.1)
(Missing)	20

Percentages are calculated among patients with a known fusion partner (*n* = 48).

### Survival outcomes

The median follow-up time was 45 months (interquartile range [IQR]: 33–58). In the unadjusted analysis, PFS for *RET*-positive patients was similar to that of the *ALK*- and *EGFR*-positive groups, whereas after adjustment, *ALK*-positive patients had the longest PFS (median 31 months, 95% CI: 22–42), and *RET*-positive patients (13 months, 95% CI: 9–19) were in line with the *KRAS*-mutated and wild-type groups (Table 3 and Figure 2 A, B). For OS, adjusted median OS was numerically longest for *ALK*-positive patients (39 months, 95% CI: 29–52), followed by *RET*-positive patients (28 months, 95% CI: 13–46), and slightly shorter for *EGFR*-positive (26 months, 95% CI: 22–29) and *BRAF*-positive patients (23 months, 95% CI: 19–30) (Table 3 and Figure 3 A, B). *ROS1*-positive, *KRAS*-mutated, and wild-type patients had numerically shorter adjusted median OS of 17–19 months. The confidence interval for the *RET* group was wide and overlapped with those of most other groups.

**Table 3:** Median OS and PFS stratified by mutational groups.

Group	Model	Median OS, months (95% CI)	Median PFS, months (95% CI)
RET	KM	NA (24–NA)	23 (12–NA)
	Cox	28 (13–46)	13 (9–19)
ALK	KM	45 (35–NA)	36 (30–45)
	Cox	39 (29–52)	31 (22–42)
BRAF	KM	15 (11–21)	12 (9–19)
	Cox	23 (19–30)	18 (15–23)
EGFR	KM	35 (32–39)	28 (25–31)
	Cox	26 (22–29)	21 (19–23)
KRAS	KM	16 (14–17)	13 (12–14)
	Cox	17 (15–18)	13 (12–15)
ROS1	KM	27 (16–NA)	19 (10–40)
	Cox	19 (8–NA)	13 (6–30)
No mutation	KM	15 (14–16)	13 (12–14)
	Cox	17 (16–19)	14 (14–15)

OS, overall survival; PFS, progression-free survival; KM, Kaplan-Meier; Cox, adjusted multivariable Cox proportional hazards model; NA, not available.

**Figure 2:**
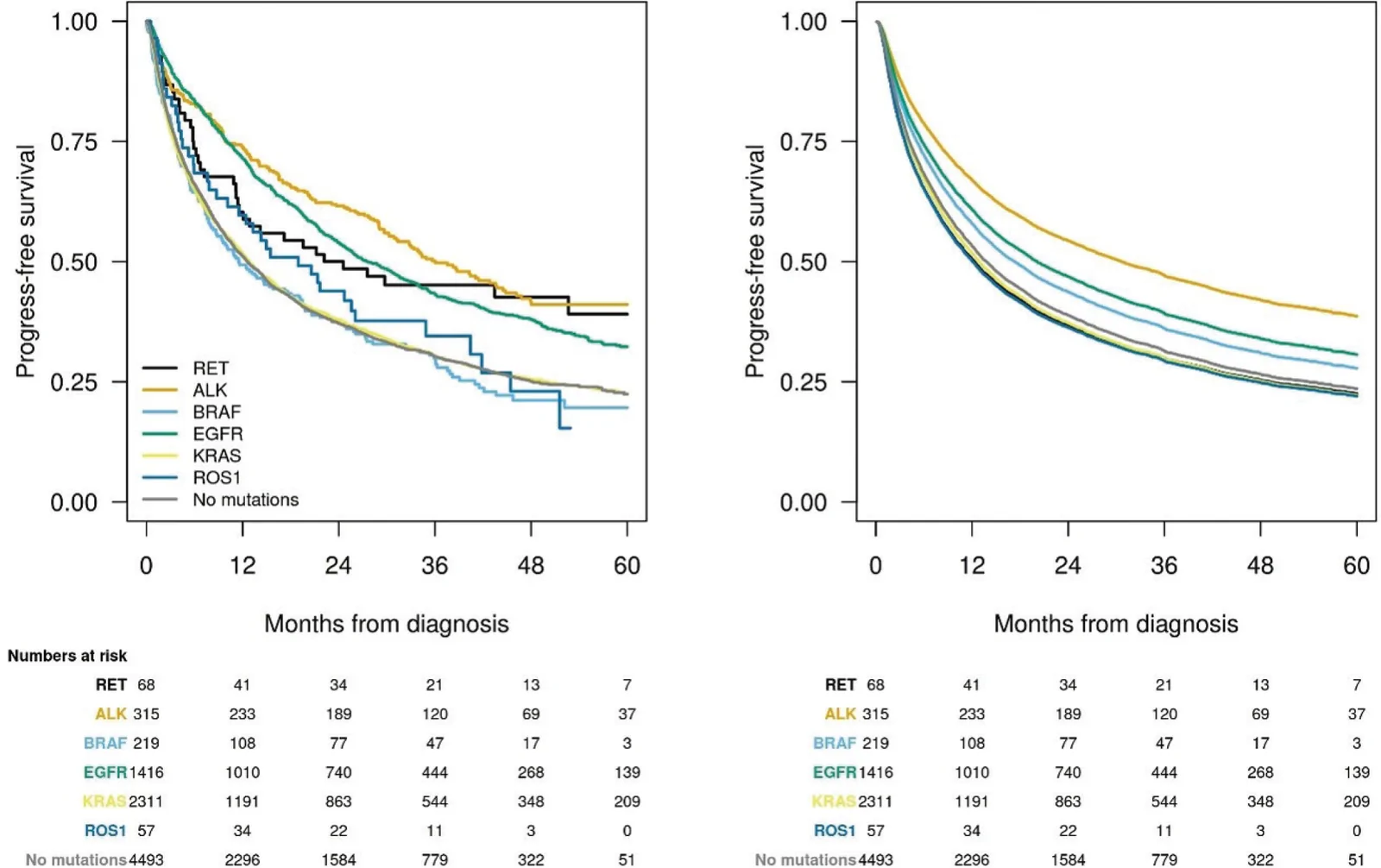
Progression-free survival stratified by mutational groups: (A) unadjusted, (B) adjusted.

**Figure 3:**
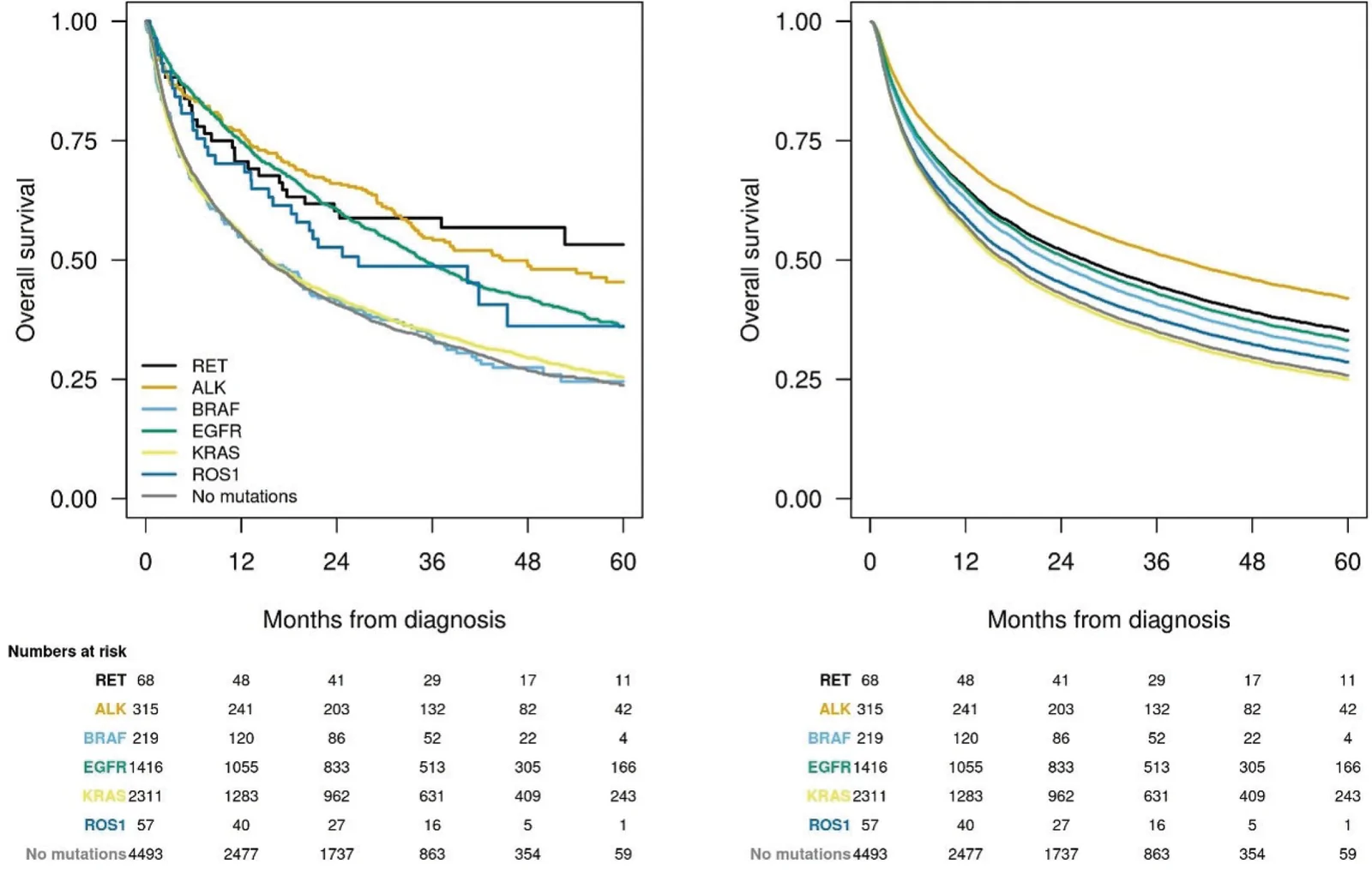
Overall survival stratified by mutational groups: (A) unadjusted, (B) adjusted.

### Therapeutic strategies and front-line treatment for RET patients

Out of 68 patients, information on first treatment was available for 56 patients. Surgery was, as expected, performed in the majority of stage I and II patients (Supplementary Table 1). Patients with stage III disease (*n* = 9) were few and received heterogeneous treatment strategies; they were therefore not included in the treatment tables, which present stage I–II (Supplementary Table 1) and stage IV (Table 4) separately. Stage III patients were not grouped with stage IV in any analysis of treatment patterns. The majority of patients with stage IV disease were given CT or chemoimmunotherapy, and only two patients received first-line RET-specific tyrosine kinase inhibitors (TKI) (pralsetinib) (Table 4). No *RET*-positive patient with stage IV disease underwent surgery as first treatment; in the overall cohort, surgery as first treatment in stage IV disease (*n* = 36) mainly comprised resection of a solitary oligometastatic disease, for example, brain metastasis. TKIs were more frequently given to the *RET* patients in the second or third line (Supplementary Tables 2 and 3) with a number of different agents, including crizotinib, lorlatinib, and cabozantinib. In total, 15 patients received selpercatinib or pralsetinib at some time during their disease course, and in the exploratory time-varying Cox model, treatment with these agents was associated with a numerically, but not statistically significant, lower risk of death (HR 0.60, 95% CI: 0.21–2.16).

**Table 4:** First treatment stage IV.

Characteristic *n* (%)	Overall 4,753	RET 38	ALK 227	BRAF 143	EGFR 822	KRAS 1,256	ROS1 40	No mutations 2,227
**First treatment**								
Chemotherapy	538 (22)	11 (39)	11 (8.1)	10 (14)	49 (9.9)	221 (30)	5 (19)	231 (24)
Immunotherapy, monotherapy	400 (16)	2 (7.1)	3 (2.2)	18 (25)	10 (2.0)	183 (25)	2 (7.7)	182 (19)
Immunotherapy, combination	514 (21)	10 (36)	8 (5.9)	13 (18)	22 (4.4)	145 (20)	1 (3.8)	315 (33)
RT	466 (19)	3 (11)	10 (7.4)	8 (11)	66 (13)	173 (24)	4 (15)	202 (21)
Surgery	36 (1.5)	0 (0)	2 (1.5)	0 (0)	5 (1.0)	12 (1.6)	0 (0)	17 (1.8)
TKI	506 (21)	2 (7.1)	101 (75)	24 (33)	344 (69)	2 (0.3)	14 (54)	19 (2.0)
(Missing)	2,293	10	92	70	326	520	14	1,261

RT: radiotherapy; TKI: tyrosine kinase inhibitor.

## Discussion

This study shows that the *RET* population seems to be similar to other NSCLC subgroups usually associated with a higher frequency of never-smokers (i.e. *ALK, EGFR*, and *ROS1*), in contrast to *KRAS* and *BRAF* mutations, which are more often diagnosed among patients with smoking history. In the *RET* cohort, almost all tumors were adenocarcinomas, and the PD-L1 pattern was consistent with what is expected in a general NSCLC population. In the entire cohort, a majority of patients presented at stage IV, with a higher proportion of liver metastases in the *RET* cohort. The most common fusion partners, *KIF5B* and *CCDC6,* are in line with other reports [16–19].

Treatment patterns in the *RET* group were as expected in the early stages, with a high surgery rate possibly due to favorable PS. In stage IV disease, the majority of patients received either CT or a combination of CT and immunotherapy as their first-line treatment, as RET-selective inhibitors were not generally available during this period. A significant proportion of *RET* patients were treated with TKIs of various kinds in the second- and third-line setting. The *RET* cohort generally had a favorable prognosis, with numerically superior OS compared to all other groups except for *ALK*-positive patients, despite not being treated with RET-selective TKIs systematically. The exploratory analysis suggests that patients who received selpercatinib or pralsetinib at some time point had numerically better survival, although the difference was not statistically significant and the result should be regarded as hypothesis-generating.

In our cohort, *RET* fusions were detected in 0.8% of all tested patients and in 1.0% of adenocarcinomas, which is at the lower end of the 1–2% reported internationally [9] but more in line with the more recent study from the Netherlands [20]. As *RET* fusion analysis was not part of all laboratories’ routine panels from the start of the study period, the frequency in Sweden is probably underestimated during the early years.

In this first nationwide Swedish cohort of patients, clinical and demographic characteristics of the *RET*-positive patients were largely consistent with those described in other real-world studies, with a dominance of adenocarcinoma histology, mainly never-smokers, and most often diagnosed at an advanced stage. One study comparing *RET*-positive and *RET*-negative NSCLC patients found that *RET*-positive patients tend to be younger, predominantly never-smokers, and have nonsquamous histology [16]. Similarly, an American cohort study [21] reported a predominance of adenocarcinomas and never-smokers, and two-thirds had metastatic disease at presentation. As in our study, rather few patients received selective RET TKIs, reflecting limited access during the study period. Median OS in the American study was 17.5 months, which is numerically shorter than the adjusted OS of 28 months observed in the current dataset, where *RET*-positive patients had outcomes slightly numerically inferior to *ALK*-driven disease but similar to those with *EGFR* mutations. A multinational analysis by Kiiskinen et al. [22] similarly supported these demographic patterns. As in the present cohort, the majority of patients initially received platinum-based CT or chemoimmunotherapy before the introduction of RET-selective inhibitors. Importantly, Kiiskinen et al. reported improved survival once selpercatinib and pralsetinib became available, highlighting the potential clinical impact of early molecular testing and targeted therapy. Comparable observations were reported in a Chinese registry study with a slightly higher proportion of female patients and never-smokers (69%). The authors observed that PFS and OS were substantially shorter among patients treated with conventional CT compared with those receiving selective RET inhibitors [18].

The comparisons with other molecular subgroups should be interpreted with caution. These groups were included as descriptive reference groups to place the *RET* cohort in the context of the contemporary Swedish NSCLC population, and they are themselves heterogeneous: *EGFR*, *KRAS*, and *BRAF* alterations comprise molecular subtypes with different prognosis and therapeutic options (e.g. common versus uncommon *EGFR* mutations, *KRAS* G12C versus non-G12C, and *BRAF* V600E versus non-V600E), which were not distinguished in the present analysis. Outcomes in the different subgroups are also strongly influenced by access to effective first-line targeted therapy. During 2018–2022, first-line TKIs were available for *EGFR*-, *ALK*-, *ROS1*-, and for *BRAF V600E*-positive disease, whereas *RET*-positive patients mainly received first-line CT or chemoimmunotherapy. Availability of first-line TKI therapy should therefore be regarded as one of the factors underlying the survival differences between subgroups. Although modern systemic treatment options were generally available throughout the study period, chemoimmunotherapy was gradually implemented during 2018. Consequently, patients diagnosed earlier in the study period may have had more limited access to chemoimmunotherapy, potentially contributing to temporal differences in outcomes, particularly among patients without actionable molecular alterations. In contrast, the benefit of immune checkpoint inhibitors in *RET*-positive NSCLC appears limited [23, 24], and the favorable outcome observed in our *RET* cohort is therefore unlikely to be explained by immunotherapy alone. Differences in access to novel treatments have been identified as a factor potentially contributing to survival differences between the Nordic countries [25]. Taken together, the observed survival differences between molecular subgroups reflect both patient selection, tumor biology and treatment availability when it comes to RET and should not be interpreted as causal.

The strengths of the NLCR data include its strong representativeness of the Swedish national population, as it covers approximately 97% of all lung cancer cases in the country, but several limitations should be acknowledged. Notably, the database does not currently capture data on ethnicity, body mass index, or socioeconomic status. The retrospective nature inherently carries risks of incomplete reporting, missing data, and potential misclassification. Data on treatment-related toxicity, which may influence treatment duration and survival, are not captured in the registry, and progression was not assessed according to formal RECIST criteria. Although the underlying NSCLC registry included nearly 9,000 patients, only a small number of *RET*-positive cases were identified, limiting the statistical power for subgroup analyses and survival comparisons. Pooling of data across countries, for example, within a Nordic collaboration, would increase the sample size. *RET* fusion analysis, although recommended, was not included in all routine NGS panels from the start of the study period, and initially there may be a bias with regard to selective testing, that is, sending a request for *RET* assessment in younger never-smokers where another oncogenic driver has not been found. Finally, the majority of patients in this cohort were treated before the systematic introduction of selective RET inhibitors, meaning the survival outcomes do not reflect today’s standard with first-line selective RET inhibition.

## Conclusion

We have presented real-world data from a population-based cohort of *RET* fusion–positive patients, compared to groups with other oncogenic drivers as well as wild-type NSCLC patients in Sweden. The *RET* fusion–positive cohort is characterized by younger age, a high proportion of never-smokers, a predominance of female patients, the adenocarcinoma subtype, and a significant presence of stage IV disease at diagnosis and an encouraging prognosis despite limited access to selective RET inhibitors during the study period, although comparisons with other molecular subgroups should be interpreted with caution.

## Supplementary Material


