# Supplementary material for: RET fusion–positive non-small cell lung cancer in Sweden: a nationwide registry study
Source: Acta Oncol. 2026 Oct 29;65:46817. doi: 10.2340/1651-226X.2026.46817 (PMC13723043; doi:10.2340/1651-226X.2026.46817)
Supplement: Supplementary file 1 [file AO-65-46817-s1.pdf]

Supplementary material has been published as submitted. It has not been copyedited, or typeset by Acta Oncologica

Supplementary Table 1 First treatment in stage I and II disease

Characteristic<br>n (%)	Overall<br>2477	RET<br>21	ALK<br>49	BRAF<br>49	EGFR<br>433	KRAS<br>673	ROS1 12	No mutations<br>1240
**First treatment**								
Chemotherapy	115 (8.2)	0 (0)	3 (10)	3 (10)	8 (3.2)	35 (7.8)	1 (14)	65 (10)
Immunotherapy, monotherapy	37 (2.6)	0 (0)	0 (0)	1 (3.3)	0 (0)	12 (2.7)	0 (0)	24 (3.8)
Immunotherapy, combination	18 (1.3)	0 (0)	0 (0)	0 (0)	1 (0.4)	8 (1.8)	0 (0)	9 (1.4)
RT	394 (28)	3 (16)	9 (31)	11 (37)	52 (21)	110 (24)	5 (71)	204 (33)
Surgery	827 (59)	16 (84)	16 (55)	15 (50)	174 (70)	284 (63)	1 (14)	321 (51)
TKI	17 (1.2)	0 (0)	1 (3.4)	0 (0)	13 (5.2)	0 (0)	0 (0)	3 (0.5)
(Missing)	1069	2	20	19	185	224	5	614

Percentages are calculated among patients with available information on first treatment. Patients with stage III disease are not shown because of small numbers and heterogeneous treatment strategies. Immunotherapy, combination: immunotherapy combined with chemotherapy and/or another immunotherapy agent. RT, radiotherapy; TKI, tyrosine kinase inhibitor.

Supplementary Table 2 Second-line therapy in stage IV disease

Characteristic<br>n (%)	RET<br>22	ALK<br>57	BRAF<br>27	EGFR<br>267	KRAS<br>316	ROS1<br>12	No mutations<br>387
Treatment							
Chemotherapy	6 (27)	8 (14)	8 (30)	75 (28)	154 (49)	3 (25)	173 (45)
Immunotherapy, monotherapy	5 (23)	2 (3.5)	7 (26)	8 (3.0)	115 (36)	0 (0)	138 (36)
Immunotherapy, combination	1 (4.5)	1 (1.8)	9 (33)	12 (4.5)	29 (9.2)	2 (17)	57 (15)
TKI	10 (45)	46 (81)	3 (11)	172 (64)	18 (5.7)	7 (58)	19 (4.9)

Percentages are calculated among patients who received the respective line of therapy. Immunotherapy, combination: immunotherapy combined with chemotherapy and/or another immunotherapy agent. TKI, tyrosine kinase inhibitor. In the *RET* group, TKIs comprised the selective RET inhibitors selpercatinib and pralsetinib as well as other TKIs (crizotinib, lorlatinib and cabozantinib).

Supplementary Table 3 Third-line therapy in stage IV disease

Characteristic<br>n (%)	RET<br>17	ALK<br>29	BRAF<br>11	EGFR<br>140	KRAS<br>147	ROS1<br>8	No mutations<br>180
Treatment							
Chemotherapy	7 (41)	6 (21)	4 (36)	62 (44)	84 (57)	4 (50)	113 (63)
Immunotherapy, monotherapy	3 (18)	2 (6.9)	3 (27)	5 (3.6)	41 (28)	1 (13)	43 (24)
Immunotherapy, combination	2 (12)	1 (3.4)	1 (9.1)	13 (9.3)	10 (6.8)	1 (13)	15 (8.3)
TKI	5 (29)	20 (69)	3 (27)	60 (43)	12 (8.2)	2 (25)	9 (5.0)

Percentages are calculated among patients who received the respective line of therapy. Immunotherapy, combination: immunotherapy combined with chemotherapy and/or another immunotherapy agent. TKI, tyrosine kinase inhibitor. In the *RET* group, TKIs comprised the selective RET inhibitors selpercatinib and pralsetinib as well as other TKIs (crizotinib, lorlatinib and cabozantinib).
